# Obeying Authority: Should We Trust Them or Not?

**DOI:** 10.1007/s12124-022-09691-7

**Published:** 2022-04-22

**Authors:** Daniel Walker

**Affiliations:** Edge Hill University, Ormskirk, United Kingdom

**Keywords:** Obedience, Power-knowledge, Authority, General Demarcation Problem

## Abstract

Researchers claim impartiality when conducting research and suggest their motives are to improve knowledge. However, when investigating the history of research into obedience to authority, propaganda and power-knowledge are present as well as emotional ties that affect the motives and methods of investigating these areas. With published work from US President Woodrow Wilson proposing obeying authority is necessary to functional societies and the Vatican displaying power-knowledge when censoring heliocentric views, it seems some researchers have ulterior motives. Although researchers like Piaget and Milgram appear to be more integral researchers, Piaget like many utilised observational methods that lack replicability, and Milgram’s family history with the events of the Holocaust pose additional issues. Therefore, considering the General Demarcation Problem, it is difficult to distinguish between science and pseudoscience, given all researchers will consider the research they conduct in the present day to be the correct way of doing so. However, adopting a critical mind as to who is conducting the research and the wider implications of who it serves and who it does not serve, would be beneficial for academia and wider society. This comes in a time where many reject the science of critical world issues such as COVID-19 and climate change.

## Introduction

Philosopher Imre Lakatos (Lakatos & Feyerabend, 1999) once reminisced on challenging Marxists on their strong views. Questioning what type of event would have to occur to alter their political and economic thoughts, stunned silence was received. This could be an example of the occurrence of cognitive dissonance, that may decelerate intellectual progression. This not only shapes how the world is perceived but the ways in which researchers attempt to gain further understanding. Just as Marxists were stunned at the thought of a world where their political beliefs would not remain constant, the same can be said for research methodologies. Khun (1962) suggested this was because scientists work within a rigid framework whereby researchers share values and beliefs, or paradigms, and described this as ‘normal science’. People have attempted to increase understanding of the world for over two thousand years however, their methods alter significantly. A crucial issue is that many researchers presume that present day methods are the best way to improve knowledge highlighting they are not exempt from cognitive dissonance. Obedience to authority has been adopted by the psychological community as a popular topic of research interest. However, the reasons as to why this area of research has been investigated differ, and therefore the conclusions ought to be inferred with caution. With present-day examples of disobeying authority such as infringement of government guidelines regarding COVID-19, this area is more pertinent than ever.

## Power of research in the early 17th -20th century

The first psychology laboratory was established in 1879 at Leipzig University. It was from then, that psychology became the dominant discipline when examining obedience to authority. Before this, many recorded documents that can be accessed provide somewhat romantic philosophical notions of the benefits of obeying authority. Whilst some papers identify obedience to authority as being an essential cog for a healthy functional society (Tuttle, 1943; Wilson, 1911) others deem it imperative for successful winning sports teams (Gettell, 1917). Without empirical evidence or data to examine, the question that once again derives is why was this propaganda published in academic books and journals of the time?

The fundamental explanation is power-knowledge. Michel Foucault is considered one of the most influential researchers regarding this phenomenon. With a background in both philosophy and psychology, Foucault was a respected researcher and claimed that power and knowledge should not be treated as two separate entities but are in-fact related. That is, knowledge is always an exercise of power and power is always a function of knowledge. Foucault (1980) used sexuality and the church as an example of power-knowledge stating that between the 17th and 20th century, the church produced knowledge of individuals’ sexual desires through confessions. He argued that through confessing these sexual desires, people began to develop a sexual identity that had not been present prior to this, an identity that, in the view of the church, had to be controlled. With biblical references that suggest certain sexual desires, such as homosexuality to be inappropriate and the church having a great influence over societal implications, the church could now use this knowledge of sexual desires provided through the confessions to control the populations’ sexual activity and perceptions of different sexual preferences.

Although the inception of Foucault’s theory of power-knowledge (1980) was only derived in the late 20th century, there are examples throughout history whereby knowledge has been utilised to not only obtain power but also to retain it. As well as the history of sexuality that Foucault (1980) highlights, the roman catholic church has also been responsible for other societal control over the development of knowledge. This is evident in the Galileo affair during the early 17th century, where the Roman Catholic inquisition tried infamous astronomer Galileo Galilei for his support of heliocentrism. Living in a region under predominant Roman Catholic influence, and during a time where the Church of England was forming, it can be considered brave of Galileo to attempt to reinterpret the Bible with his astronomical observations and heliocentric conclusions (Langford, 1992). Having made his views clear, however, Galileo was ordered by Pope Paul V to abandon these notions that the Earth and other planets orbit the sun as they oppose holy scripture (Finocchiaro, 2014). For ten years Galileo obliged, until the election of a new Pope that had condemned his earlier discipline from the church. The Church’s elitist authoritarianism towards Galileo and heliocentrism at this time had desperate undertones with a fear of increased knowledge leading to secularisation and in turn a forfeit of power. Censoring the work of Galileo, the Church controlled the knowledge that was available to the public at that time. Only allowing publications and merit to those that supported the biblical, geocentric views of the Vatican in turn led to the retention of power in this period.

This is also evident with the first philosophical notions of obedience to authority in the early 20th century. As highlighted, prior to any objective psychological examinations of this behaviour, there were various non-empirical suggestions that obedience is necessary for a productive functional society (Tuttle, 1943; Wilson, 1911). Power-knowledge provides a good explanation as to why this propaganda would be published in trusted sources. Wilson (1911), soon to become President of the United States Woodrow Wilson, referred to obedience to authority as being integral to a functional society with the paper being published just two years before his presidential election victory, and whilst governor of New Jersey. Published in The American Political Science Review, a leading political journal within the country, this could be construed as a form of propaganda, to develop power over the masses. With the notion that individuals are led to believe that the power of the few over the many is to their advantage (Lakatos & Feyerabend, 1999), there are undertones that the references of obedience to authority are utilised with similar motivations of the Roman Catholic Church in the 1600’s. However, in the interests of furthering research, fortunately a more epistemological and systematic method of examining obedience to authority was adopted in the 20th century.

## Piaget’s impact on objectively examining obedience to authority

Swiss psychologist Jean Piaget originally had a background in philosophy due to his godfather’s urgings to study the fields of philosophy and logic (Snowman & Biehler, 2003). It was only upon emigrating to France in the 1920’s where his interest in psychoanalysis developed, accompanied by experimental methods. His work consisted predominantly of the cognitive development of children which led to studying infant morality (Piaget, 1932). One of Piaget’s basic principles regarding morality in children was the creation of their own conceptions of the world, in that they are not genetic or adopted through social observation of adults. That is, infants develop their own understanding of justice, fairness and equality based on how they perceive the world and through social interaction with peers, supporting Kantian theory (Kant, 1999). Interestingly, Piaget (1932) reported that when obedience to authority and equality are brought into conflict, the child is always in favour of obeying the authoritative figure of the adult and disregarding their own moral compass. This work can be considered a pioneering aspect of literature into obedience to authority that has led to an abundance of psychological research throughout the 20th and into the 21st century (Brief et al., 2000; Gridley & Jenkins, 2017; Laupa & Turiel, 1986; Milgram, 1965).

Piaget’s original claims were based on a relatively small sample of French children in a controlled experimental condition, and therefore it would not be plausible to accept the notion that all children’s moral reasoning is heteronomous before ten years of age where it then becomes autonomous, despite others reporting similar findings (Lerner, 1937). Instead, Piaget can be credited for providing the basis for further research such as Kohlberg’s stage theory of moral development (Kohlberg, 1969) which has been widely accepted by the psychological community (Malinowski & Smith, 1985; Murphy & Gilligan, 1980). While critics argue that both Piaget and Kohlberg severely underestimated children and their cognitive processes (Leonard & Archer, 1989; Narvaez, 2005; Rubin & Trotter, 1977), suggesting that stages of moral development occur earlier than five years of age (Cushman et al., 2013; Gray et al., 2004), the pioneering work of Piaget provided a precedent of further research into an unscathed area of psychology. Following research into children, it was of interest to many whether urges to obey authority would continue into adulthood, a decision possibly influenced by real life events (Mastroianni, 2002).

## Milgram, World War Two, and the Holocaust

The most prominent study regarding obedience to authority (Blass, 1999) is Milgram’s experiment (Milgram, 1963) which is considered one of the most popular in psychology (Slater et al., 2006). Winning the AAAS Prize for Behavioural Science Research in 1964 for his work on social aspects of obedience, Milgram’s reputation was projected to soar. Ethical debates that surrounded his work led to Milgram failing to secure a tenure position at Harvard University, despite the controversial experiment taking place at Yale. However, disregarding the ethical implications, this study supports the conclusions of Piaget’s earlier work, finding that although the sample were aware from a very young age that hurting others is against their moral code, they abandon these beliefs when following the instructions of an authoritative figure (Milgram, 1963).

Scientific research often follows the normal science structure that an idea forms the basis of a research question which is then tested and reported on (Khun, 1962). It would appear this is the case with Milgram wanting to study obedience to authority, following on from the work of Piaget (1932) and then McGranahan (1946) who examined the differences between obedience to authority in German and American samples. However, raised in a Jewish household, to refugee parents, Milgram was directly affected by the Holocaust. Surviving family members that bore concentration camp tattoos even took sanction at their new home in New York during the second world war (Fermaglich, 2007). Additionally, during adulthood, the worldwide media coverage of the Adolf Eichmann trial persisted. The former Nazi concentration camp leader had been charged with fifteen crimes, including war crimes and crimes against humanity in 1960 (Arendt, 1994). Two years later Eichmann was found guilty and subsequently executed, however observers of the trial reported Eichmann’s remarkable normalcy whilst attempting to hide behind the fact that he had committed no crime and was simply following orders. It was insisted that he and others were bound by an oath of loyalty to Hitler which was the same superior orders defence used by many defendants in the Nuremberg trials a decade earlier (Cesarani, 2005). The aftermath of the execution led to increased media coverage and renewed interest of the second world war and the holocaust (Cesarani, 2005) and Milgram was no exception. There were even mentions of Nazi behaviour in several pieces of his work into obedience (Milgram, 1963; 1965). Although initial ideas form a significant basis of psychological research, how much influence do real life events have on researchers?

## General Demarcation Problem

The rationale behind Milgram’s cognition raises questions about the general demarcation problem. Where previously there were philosophical propogandist notions of obedience to authority being essential to a functional society (Tuttle, 1943; Wilson, 1911), following Piaget’s example, Milgram was now willing to utilise more testable techniques to examine obedience. The general demarcation problem is the debate between science and non-science and is considered one of the most important issues in the philosophy of science and therefore social science (Resnik, 2000). There are various criterion of the demarcation problem including, logical positivism. Logical positivism supports verificationism and asserts that only statements that can be verified through empirical observation are cognitively meaningful. Popper (1963) added that to be considered a science, the theories and hypotheses must be testable (Resnik, 2000). This was referred to as falsifiability, another branch of the demarcation problem that opposes verificationism (Thagard & Zalta, 2008).

From this, it can be considered phenomenological that obedience to authority is researched today. The non-empirical suggestions in the early 20th century (Tuttle, 1943; Wilson, 1911) had tones of propaganda and therefore would be considered as ‘non-science’ if taking a Popperian standpoint, highlighting the demarcation problem among this area of research. Following this, Piaget’s (1932) observations of children’s responses to obeying authority and ignoring their own moral code could be said to agree with the viewpoint of logical positivism. The conclusions that when obeying authority and equality are brought into conflict, children are always in favour of obeying the authoritative figure and disregarding their own moral beliefs, can be considered as logical empiricism. The empirical observation of the researchers provides a cognitive meaning to the statements, in this case conclusions, of the research.

Milgram’s study into obedience to authority (1963) can be considered, unlike Piaget’s work of children’s cognitions, an example of falsifiability. The fundamental difference between logical positivism and falsifiability is that the latter has the capacity for theory to be proven wrong. As Milgram reported in his study the sample used and the exact methodological procedure of the experiment this allows for replication and in turn criticism of the findings, something that was impossible with Piaget’s observations. This has become evident with numerous studies highlighting the lack of validity of Milgram’s research (De Vos, 2009; Fjellman, 1976) with some even suggesting the results were fabricated (Perry, 2013). From this, the demarcation problem makes it difficult to differentiate between science and pseudoscience. Where most would agree that there is a clear distinction between the work of Wilson (1911) and Tuttle (1943) being pseudoscience and Piaget (1932) and Milgram (1963) being science, it is difficult to suggest logical positivism should not be considered as scientific as other criterion of the demarcation problem exist such as falsifiability of data.

## Why conduct the research, who does it serve, and who does it not serve?

From the literature examined, the reasons behind conducting research vary. Whereby some researchers share the paradigm that the sole purpose of research is driven by the philosophy of advancing science and improving knowledge (Eisenberg, 1987; Owen et al., 2012), it is evident that the driving force behind some research alters. From the work of Wilson (1911) we can deduct that there is a political agenda involved, utilising the platform he established during his academic career during his presidential campaign. In contrast, the work of Milgram taken on face value could be considered normal science (Khun, 1962) as it can be seen to progress former aspects of the literature (McGranahan, 1946). However, there is the argument that Milgram’s real-life experiences question the motives and positionality of this research. Milgram’s research, although valuable in its findings into the human cognitive function of obedience (Milgram, 1965), can be questioned for providing former Nazi’s with an excuse for their behaviour during the holocaust as well as diminishing responsibility. Having considered the reasons for conducting research and who it may serve, there is cause to acknowledge those that it may not be beneficial to. As Germany and former Nazi’s now had the excuse of an agentic state (Nissani, 1990), this research can be considered as not serving victims of the holocaust by defending the perpetrators, something that Milgram was heavily criticised for given his background (Mastroianni, 2002).

## Present-Day Implications

It is important to be aware of science and pseudoscience, and how this could influence the masses. This is especially relevant in the present day, given the speed at which misinformation is spread online, from climate change (Allagaier, 2019) to COVID-19 (Li et al., 2020). It is vital that the present-day public do not succumb to the cognitive dissonance errors of previous generations, and to be aware of how people in positions of power, are able to use different sources to push their agenda. Rather, a critical approach to research should be adopted, understanding the role that power-knowledge could have, the reasons for the research being conducted, and who is conducting it. This is particularly relevant to anti-vax sentiment and COVID-19 belief with obeying authority.

The General Demarcation Problem is particularly pertinent in the information and misinformation regarding COVID-19. Interestingly, logical positivism has been adopted by many during the COVID-19 pandemic when looking to scientific knowledge to inform their behaviour. However, this has been threatened as many have not subscribed to this, actively opposing scientific knowledge in fear of propaganda from worldwide governments and the mainstream media. This may have been exacerbated by political shortfalls such as Downing Street parties by the United Kingdom government as well as the former Health Secretary disobeying the guidance that they had set themselves. Despite conference briefings including Chief Medical Officer for England, Professor Chris Whitty, perhaps the political undertones of their delivery cast doubt into some minds regarding the validity of these claims. Therefore, there is anti-vaccine sentiment that questions what is science and pseudoscience regarding COVID-19 due to political inclusion within science. Interestingly, this demonstrates a clear shift in obedience towards authority as Wilson (1911) received little backlash from his academic publication. By contrast, the now Prime Minister of the United Kingdom receives large criticism and scrutiny from the British public on social media websites such as Twitter.

Many also oppose climate change due to authority informing them of its dangers and encouraging behaviour change within the general population. The COVID-19 lockdowns and climate change issues share a similarity in their hardship. Many report the governmental restrictions imposed due to the onset of the pandemic as being extremely difficult to their mental health (Banks & Xu, 2020). Additionally, many also suggest that climate change is an extremely difficult issue to address due to its cumulative effect (Clerici et al., 2019; Murray et al., 2015). The similarity here may be their difficulty. Although those that refute COVID-19 or climate change may cite reasons such as political power and restricting societal freedoms as their motivations, it could be that they are afraid of the science. Rather than accepting the science as true and taking the hard steps in tackling huge societal hardships like the two examples above, many may find it easier to reject the science, regardless of its rigour.

Here we develop a new underpinning of the General Demarcation Problem. Not only is there the issue of determining the difference between science and pseudoscience, but there is also the issue of the public’s right to reject the science. While in a democratic society everyone has the right to their own opinion, this liberty to reject scientific evidence has huge implications on science itself, health, and the future of our planet. The cognitive dissonance that exacerbated the Marxists stunned silence when Lakatos confronted their beliefs may be a key reason for challenging scientific evidence for COVID-19 and climate change.

## Conclusions

When reviewing the literature as well as various historical events, it is fair to conclude that ideas are rarely novel in the context of research in social science and obedience to authority is no exception. This questions Kuhn’s (1962) notion of ‘normal science’ as it is evident that propaganda was utilised in part of the early 20th century, highlighting a different motive for conducting research. As well as this, the link between knowledge and power becomes clearer with various examples highlighting the association between the two entities and the ways in which they have been manipulated. With the links between power and knowledge proving increasingly evident, this amplifies how power can also determine what society believes to be true, and in turn censoring and controlling the knowledge of the masses (Lakatos & Feyerabend, 1999). The general demarcation problem and truth can also be perceived as interacting bodies as the constant evolution of what is and what is not a science can develop power by the disregarding of another. It is interesting to examine the ways that obedience to authority has been shaped in the literature over the past 100 years. As many modern-day researchers would stand by Kuhn’s suggestion of ‘normal science’ it is noteworthy that the inception of obedience to authority within testable research was inspired by real life events. Additionally, the role of power-knowledge and obedience to authority may be more relevant than ever, given the impact that this may have on current issues such as compliance to COVID-19 restrictions and climate change action.

## Data Availability

Data sharing not applicable to this article as no datasets were generated or analysed during the current study.
